# Interface Characteristics of Neat Melts and Binary Mixtures of Polyethylenes from Atomistic Molecular Dynamics Simulations

**DOI:** 10.3390/polym12051059

**Published:** 2020-05-06

**Authors:** Sanghun Lee, Curtis W. Frank, Do Y. Yoon

**Affiliations:** 1 Department of Chemistry, Gachon University, Seongnam 13120, Gyunggido, Korea; sanghunlee@gachon.ac.kr; 2 Department of Chemical Engineering, Stanford University, Stanford, CA 94305, USA; curt.frank@stanford.edu

**Keywords:** polymer thin films, polymer surfaces, surface tension, surface segregation, polymer mixtures, atomistic simulation

## Abstract

Molecular dynamics simulations of free-standing thin films of neat melts of polyethylene (PE) chains up to C_150_H_302_ and their binary mixtures with n-C_13_H_28_ are performed employing a united atom model. We estimate the surface tension values of PE melts from the atomic virial tensor over a range of temperatures, which are in good agreement with experimental results. Compared with short n-alkane systems, there is an enhanced surface segregation of methyl chain ends in longer PE chains. Moreover, the methyl groups become more segregated in the surface region with decreasing temperature, leading to the conclusion that the surface-segregation of methyl chain ends mainly arises from the enthalpic origin attributed to the lower cohesive energy density of terminal methyl groups. In the mixtures of two different chain lengths, the shorter chains are more likely to be found in the surface region, and this molecular segregation in moderately asymmetric mixtures in the chain length (C_13_H_28_ + C_44_H_90_) is dominated by the enthalpic effect of methyl chain ends. Such molecular segregation is further enhanced and dominated by the entropic effect of conformational constraints in the surface for the highly asymmetric mixtures containing long polymer chains (C_13_H_28_ + C_150_H_3020_). The estimated surface tension values of the mixtures are consistent with the observed molecular segregation characteristics. Despite this molecular segregation, the normalized density of methyl chain ends of the longer chain is more strongly enhanced, as compared with the all-segment density of the longer chain itself, in the surface region of melt mixtures. In addition, the molecular segregation results in higher order parameter of the shorter-chain segments at the surface and deeper persistence of surface-induced segmental order into the film for the longer chains, as compared with those in neat melt films.

## 1. Introduction

In recent years, various properties of surfaces and thin films of polymers have been investigated by various experiments, computer simulations, and theoretical approaches. Such investigations showed very important characteristics of polymer surfaces and interfaces, quite different from those of the bulk phase, which are unique to long-chain molecules. Therefore, understanding the surface/interface characteristics of polymers presents new challenges and opportunities to gain scientific insights into long-chain molecules subject to the spatial confinement in surfaces and interfaces. Moreover, understanding the surface characteristics of polymers is very important for many practical polymer applications such as thin-film coatings, adhesives, lubricants, polymer composites, organic optoelectronics, etc.

In this regard, it is known that the unique properties of polymer surfaces/interfaces are determined by the structure and dynamics within the depth of a few nanometers. This renders the molecular simulation methods ideally suited to investigate the structure-properties relationships of polymer surfaces/interfaces in greater details than other techniques. Therefore, many computer-simulation studies have been performed to understand the structural, thermodynamic, and dynamic properties of surfaces and thin films of polymeric materials [1,2,3,4,5,6,7,8,9,10,11,12,13,14,15,16,17,18,19,20,21]. 

Atomistic Monte Carlo (MC) simulation and molecular dynamics (MD) simulation are now well-known computational methods for thin film studies at the atomistic/molecular level. Mansfield and Theodorou first reported the atomistic MC simulation of free-standing polypropylene thin films [1]. In the free surface, the density inhomogeneity gives rise to an anisotropy in the mechanical stress tensor, resulting in surface tension which is one of the most important interfacial thermodynamic properties. Harris first calculated the surface tension of n-C_20_H_42_ liquid, employing the MD simulation method for a united atom model [2].

Polyethylene (PE) is one of the most popular polymers for both scientific studies and practical applications. Because of its simple chemical structure and the absence of electrostatic interaction, many MD simulation studies have been performed on PE thin films as well as its bulk phase. However, previous studies [2,3] on PE surfaces have been limited to neat liquids of short chains. In this work, we report the results of MD simulation study of thin films comprised of long PE chains, up to C_150_H_302_, and their binary mixtures with short n-alkanes, n-C_13_H_28_. This will provide us with an insight to understand various structural, thermodynamic, and dynamic properties of surfaces and thin films of polydisperse polymer systems of general interest.

## 2. Simulation Methods and System Specifications

The previous work of Chang et al. dealt with MD simulations of relatively short n-alkane chains (C_13_H_28_) [3]. In this work, we perform simulations in a similar manner on two neat melts, which contain longer polyethylene (PE) chains of C_44_H_90_ and C_150_H_302_, respectively, and two melt mixtures comprised of these PE chains with short C_13_H_28_ chains, i.e., (C_13_H_28_ + C_44_H_90_) and (C_13_H_28_ + C_150_H_302_). Each mixture was chosen to have nearly equal weight fractions in order to compare their structural and thermodynamic characteristics solely based on the chain length.

For the computer simulation of PE and hydrocarbons, many force fields have been developed both with explicit atom (EA) [22,23,24,25,26] models and united atom (UA) models [27,28,29,30]. The EA model considers each hydrogen atom as an interaction site as well as carbon atom, and the computational cost is thus much higher than with the UA model. It was demonstrated in the previous work that there is no noticeable difference between the simulation results with the EA and UA models of the predicted structural/thermodynamic characteristics of free melt surfaces of C_13_H_28_ [3]. Therefore, for long PE chains considered in this work we employ only the UA model to achieve better statistics of simulation results, which require much longer simulation times for long PE chains. In the UA model adopted here, the nonbonded interaction parameters of methyl (chain end) and methylene groups were tuned to reproduce the experimental melt densities of C_13_H_28_ and C_44_H_90_ [30].

Since the details of the simulation method on thin melt films were fully described in the previous work [3], we briefly mention some important features of the present simulations. We used Nose’s extended system method [31] to maintain the temperature at a desired value. This method introduces a virtual coordinate to control heat flow into and out of the system, and the system samples the canonical ensemble in equilibrium. The fifth-order Gear predictor-corrector algorithm [32] was used to integrate the equations of motion with a time step of 0.5 fs. While a shorter time step would give a less systematic drift of the extended Hamiltonian, the time step of 0.5 fs was sufficient to give reliable simulation results for the UA model with bond stretching. The Lennard-Jones potential for intermolecular and nonbonded intramolecular interactions was cut off at 9 Å, and the standard long-range corrections to energy and pressure were taken into account during the simulation [3].

Among various equilibrium properties, the specific volume from the fixed number of molecules at fixed pressure and temperature (NPT) MD simulation of the bulk-melt phase, the surface tension from the fixed number of molecules at fixed volume and temperature (NVT) MD simulation of the melt surface, and the distribution of the end-to-end distance of polymer chains were investigated in detail. The accumulated average of the specific volume converges rapidly within a few hundred picoseconds. However, calculating the surface tension requires much longer simulation times to obtain reproducible results, e.g., a few nanoseconds even for a short alkane system, because the surface tension is determined from the difference between the different components of the virial tensor. In this work, we carried out simulations for 10 nanoseconds on the neat melt films of C_44_H_90_ (ca. 40 Å thickness with 30 chains in the simulation box) and the mixture of C_13_H_28_ and C_44_H_90_ (100 and 30 chains, respectively), and for 50 nanoseconds on the neat melt films of C_150_H_302_ (ca. 50 Å thickness with 20 chains in simulation box) and the mixture of C_13_H_28_ and C_150_H_302_ (228 and 20 chains, respectively).

**Figure 1:**
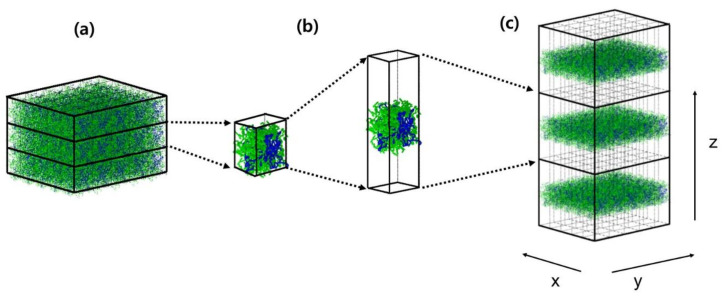
Simulation scheme to prepare a thin film: Equilibrium bulk phase with periodic boundary conditions (**a**), elongation along one (*z*) direction (**b**), and thin films with periodic boundary conditions (**c**). This snapshot was captured during simulation of the mixture of C_150_H_302_ (green) and C_13_H_28_ (blue).

Prior to performing a thin film simulation, simulation on the bulk-melt phase was first carried out in order to obtain PVT properties from NPT MD simulations performed for a cubic simulation box with periodic boundary conditions (Figure 1a). For this purpose, an initial NVT simulation with 10 × 10 × 10 nm periodic box that contained the predetermined chain length/number of PE chains as described above were carried out at 1000 K for 500 ps, and then cooled to a preset temperature under NPT simulation condition to obtain equilibrated melt system at each temperature separately. After cooling, to bring the systems to equilibrated states, they were simulated for 10 ns under NPT ensemble. The equilibration was checked by confirming that the accumulated time-averaged density remained constant and the autocorrelation function of the chain end-to-end vector converged to zero. After the bulk phase was equilibrated, the simulation box was elongated along the z-direction by about 3 to 4 times while keeping the coordinates of the atoms unchanged (Figure 1b), which generates free surfaces at both sides of the thin film. Using these coordinates as input, NVT MD simulations were performed with periodic boundary conditions in all directions (Figure 1c). The rectangular parallelepiped simulation box should have a sufficiently long separation in the z-direction such that interactions between molecules on the opposite surfaces separated by the vacuum become negligible. The evaporation of molecules into vacuum does not occur, since the vapor pressure of PE chains is negligible at the temperature of simulation. The equilibration of thin films was confirmed by monitoring the surface density profiles and the long-range correction term of the total surface tension, as explained in detail previously [3]. An average over three independently generated starting sample configurations were employed for all the data reported in this work.

The surface tension is calculated from the corresponding microscopic expression given by [33,34]

(1) $$\gamma =\frac{1}{2L}\langle 2P_{zz}-P_{xx}-P_{yy}\rangle =\frac{1}{2A}\langle 2W_{zz}-W_{xx}-W_{yy}\rangle$$

In Equation (1), $P_{\alpha \alpha }$ is the diagonal component of pressure tensor, and L is the size of the square surface. The surface tension is rewritten in terms of the virials in the last expression where A is the total surface area, and $W_{\alpha \alpha }$ is the α-component of the atomic virial tensor given by:

(2) $$W_{\alpha \alpha }=\sum_{i}^{N}\sum_{a\in i}^{m}{\left(\vec{f_{i}^{a}}\right)}_{\alpha }{\left(\vec{r_{i}^{a}}\right)}_{\alpha }$$

where N is the number of molecules, m is the number of atoms in a molecule, ${\left(\vec{f_{i}^{a}}\right)}_{\alpha }$ is the α -component of the force exerted on atom a of molecule i, and ${\left(\vec{r_{i}^{a}}\right)}_{\alpha }$ is the α-component of its position vector.

The contribution of the long-range correction term for the van der Waals interaction to the surface tension is not negligible [35]. This term can be evaluated by using the Kirkwood–Buff equation [33] for the region beyond the cut-off and assuming the pair correlation function in this region to be unity. In addition, due to the free surface, the local density around an atom is asymmetric along the (thickness) z-axis. Hence, a self-consistent correction term to the force in the z-direction was taken into account by employing the density profile. The detailed equations for such long-range corrections for the surface tension and the force were presented previously by Chang et al. [3].

## 3. Results and Discussion

### 3.1. Pure Melts of C_44_H_90_ and C_150_H_302_

**Figure 2:**
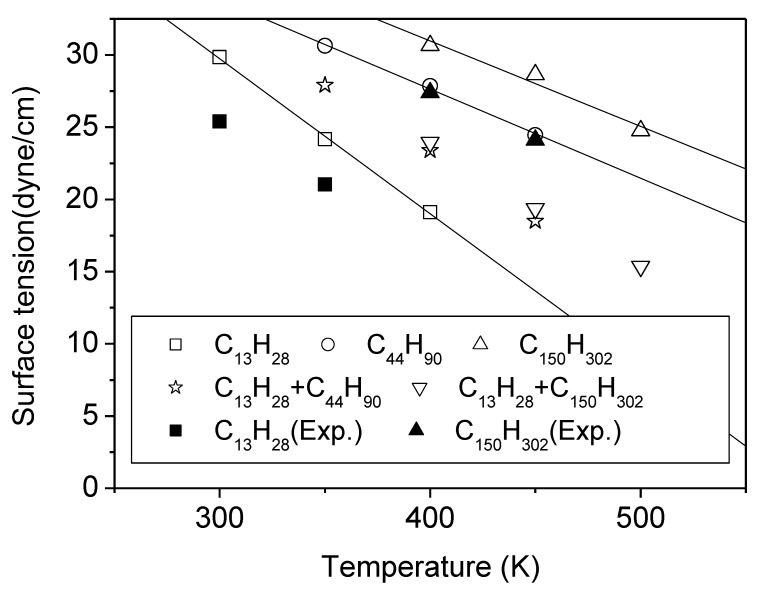
Surface tensions of polyethylene films. The open symbols are obtained from molecular dynamics (MD) simulation and the closed symbols are experimental results from references [36,37]. The simulation results of C_13_H_28_ are taken from Chang et al. [3].

In Table 1 and Figure 2 are shown the surface tensions of the PE melts calculated from MD simulations, together with the available experimental surface tension results [36,37] as denoted. The value of the surface tension increases with increasing chain length and also with decreasing temperature. The former arises from higher cohesive energy due to denser packing caused by the reduced number of chain ends and the latter from denser packing at lower temperature, as indicated by the melt specific-volume results in Table 2. The calculated values of the surface tension are somewhat larger than the experimental values with deviations of 10% to 15%. These differences are much smaller than that in the previous work of Harris [2], and are consistent with the previous results [3], indicating that the main cause is most likely to be the consideration of only pairwise interaction terms, thereby ignoring three-body and higher order interactions, in estimating the non-bonded interactions.

**Figure 3:**
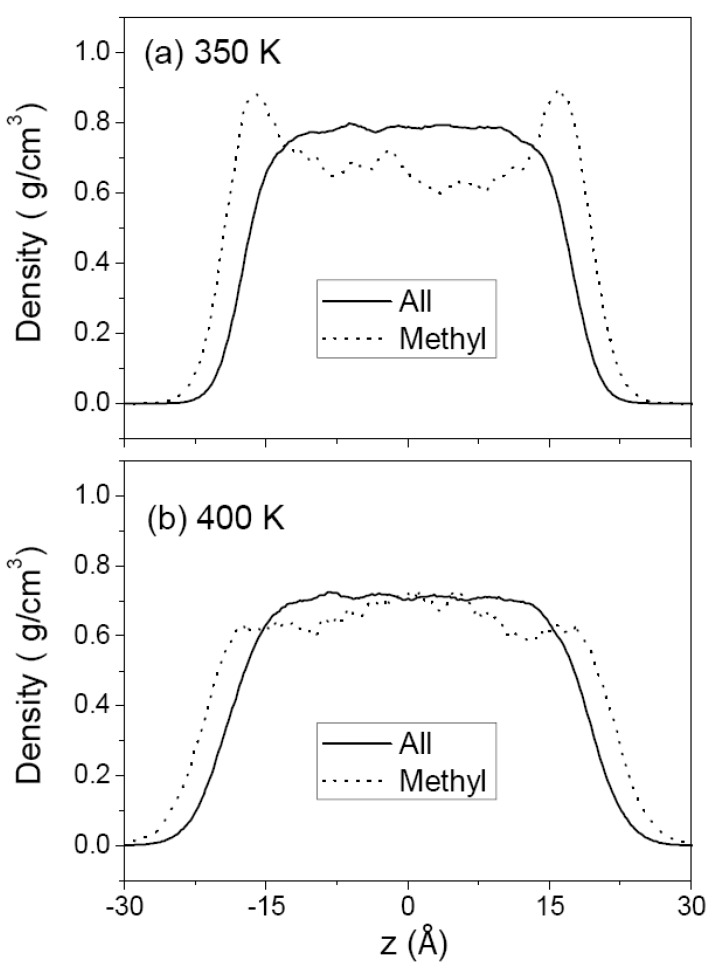
Density profiles of C_44_H_90_ melt film obtained from MD simulation at 350 K (**a**) and 400 K (**b**), along the film thickness direction (*z*) with the film center at *z* = 0. The solid line is the density of all atoms, and the dotted line is the density of methyl groups. The latter is normalized to the former for comparison by taking into account the relative total populations.

**Figure 4:**
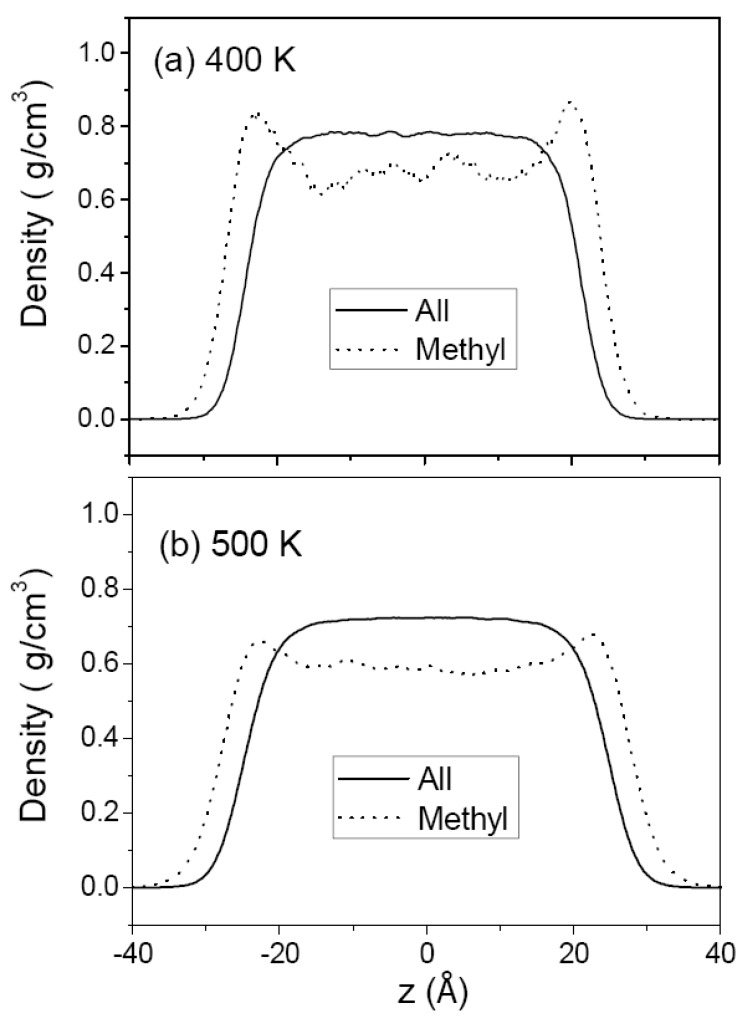
Density profiles of C_150_H_302_ melt film from MD simulation at 400 K (**a**) and 500 K (**b**); see the caption for Figure 3.

Figure 3 and Figure 4 show the density profiles of C_44_H_90_ at 350 K and 400 K, and those of C_150_H_302_ at 400 and 500 K, respectively, along the film thickness (*z*) direction with the film center at *z* = 0. For comparison, the density of methyl chain-end groups is normalized to the density of all atoms by taking into account the relative total populations. The overall density profile of all atoms has a flat bulk region and shows monotonically decreasing behavior in surface regions. Comparison of (a) and (b) in Figure 3 and Figure 4 shows the variation of the thin film structure of polyethylene with temperature. The increasing thickness of the melt film with increasing temperature is due to the thermal expansion and hence the bulk region has lower density at higher temperature. The interfacial thickness, as determined from the slope of the density profile in the surface regions, is increased when the temperature is increased, as expected.

In contrast, the density profiles of the methyl chain-end groups do not show such a simple behavior. In Figure 3a (or Figure 4a), it is seen that the methyl groups are much more likely to be found in the surface regions while they are depleted in the middle bulk-like region. At a higher temperature, the peak height in the density profile of methyl group is considerably reduced as shown in Figure 3b (or Figure 4b), consistent with the simulation results of chain-end segregation in thin polystyrene films [17]. Figure 3b and Figure 4a compare the density profiles of C_44_H_90_ and C_150_H_302_ at the same temperature of 400 K, demonstrating that the chain-end segregation becomes more pronounced in longer PE chains.

Several theoretical and experimental studies have discussed the surface segregation results of chain ends observed in the liquid/vapor and liquid/solid interfaces [17,38,39,40,41,42]. In our simulations we used a smaller energy ε parameter of the Lennard-Jones potential for the methylene group than that for the methyl group, whereas the size parameter σ is the same. Therefore, simply on the basis of different strengths in attractive energies of methylene vs. methyl groups, one would expect methylene groups to be segregated in the surface so as to minimize the enthalpy of the system, contrary to the simulation results. On the other hand, if there were no difference between the methyl and methylene groups, as in an idealized lattice-chain or pearl-necklace model, the chain-end segregation would be driven to minimize the loss of conformational entropy of polymer chains, which is a well-accepted theoretical explanation. It is noticed, however, that if this argument for the end-group segregation in terms of the entropy loss is to be valid, the segregation would be enhanced with increasing temperature as is dictated by the basic thermodynamic relationship (G = H − TS). On the contrary, our simulation results show that the increase in temperature reduces the surface-segregation of chain ends. This suggests that the major reason for the chain-end segregation is not attributable to the entropic effect, but rather to the enthalpy (or energy) effect. The problem illustrated here arises from applying idealized chain models to understand polymer surfaces and interfaces and is easily reconciled with the atomistic model of PE chains, as described below.

**Figure 5:**
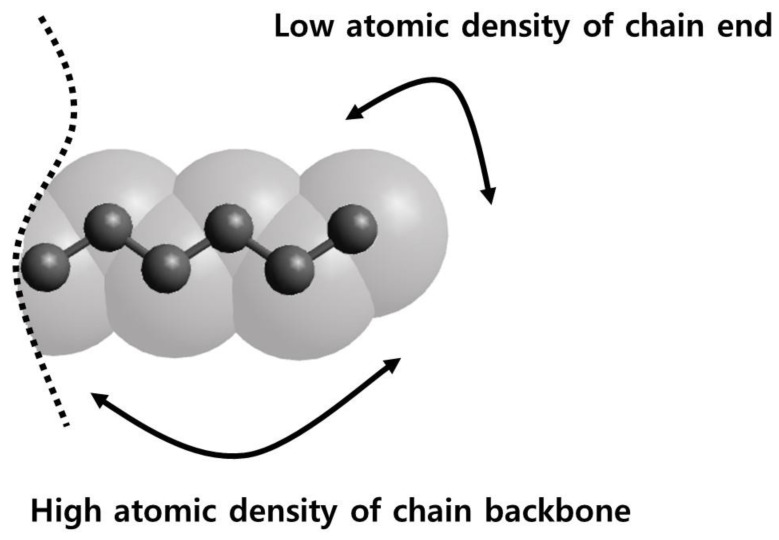
Schematic representation of the terminal portion of polyethylene (PE) chains by the ball-and-stick (connected black circles) and space-filling (silver-white) models; the black dotted line represents the remainder of a PE chain and the black arrows highlight the chain-end region vs. the backbone-like region.

The (Lennard–Jones) diameter of methylene and methyl group (~4 Å) is much larger than the C–C bond length (1.54 Å) such that the space occupied by each group overlaps considerably with its neighbors. As shown in Figure 5, the chain backbone region consisting of methylene groups has a higher packing (number) density of atoms (and, thus, a higher cohesive energy density) than the methyl chain-end region, resulting in a large difference in cohesive energy density per CH_2_ (0.312 kJ/cm^3^) vs. CH_3_ (0.122 kJ/cm^3^) [43]. From this viewpoint, the segregation of the methyl groups at the surface arises mainly from minimizing the enthalpy by pushing out the low cohesive-energy-density moiety to the surface. There is also an influence of chain length on the surface-segregation results of methyl groups. As seen in Figure 3b and Figure 4a, the methyl segregation in melt films of C_150_H_302_ is more pronounced than that in melt films of C_44_H_90_ at the same temperature. This is consistent with the enthalpy-driven effect since the atomic density is higher in C_150_H_302_ melts (Table 2) so that the driving force to push out the lower cohesive-energy-density moiety to the surface is greater, as compared with C_44_H_90_ melt films.

**Figure 6:**
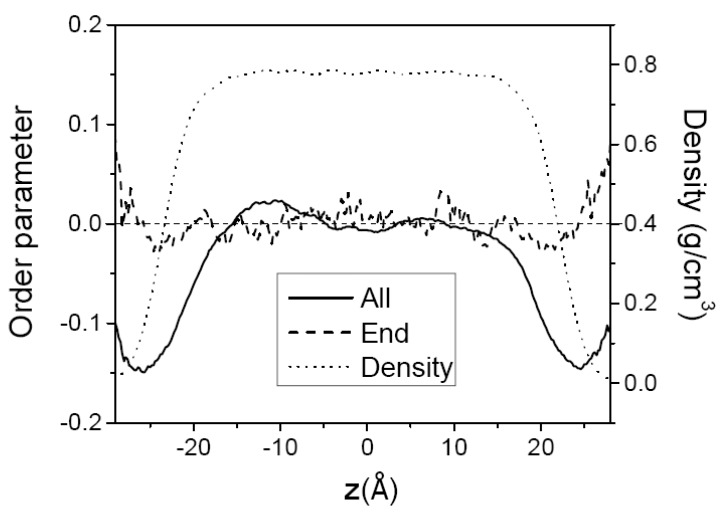
The order parameter of C_150_H_302_ melt film at 400 K along the film thickness direction (*z*) with the film center at *z* = 0. The solid line is for all segment vectors, and the dashed line is for the endmost vectors. The overall density profile of the film is shown by the dotted line.

The orientational order parameter is used to investigate the conformational behavior of chain molecules, defined as:

(3) $$P(z)=\frac{1}{2}\left(3\langle \cos^{2}\theta \rangle -1\right)$$

where *z* is the location of the mid-point of the chain-segment vector and θ is the angle between the *z*-axis normal to the film surface and a chain-segment vector joining two carbon atoms separated by two intervening bonds. The value of the order parameter is $-\frac{1}{2}$ when the chain segments are perfectly aligned in the direction parallel to the surface, 1 when they are perpendicular to the surface, and 0 when they are randomly oriented. Figure 6 shows the segment order parameter in C_150_H_302_ films at 400 K. The order parameter of all segment vectors (mostly backbone segments) is negative at the surface, and is close to zero in the middle region, showing that all segment vectors tend to favorably orient in the direction parallel to the surface in the surface region while they are randomly orientated in the middle bulk-like region. The order parameter of the endmost segment does not show any discernible orientational preference, since its value is close to zero over the whole film within the simulation uncertainty. This implies that the orientational/conformational freedom of the chain ends are hardly affected by the constraints of the film surface. A similar behavior of segmental orientational order was also observed for C_44_H_90_ not shown here.

### 3.2. Mixtures of PE Chains with C_13_H_28_

Two PE mixtures with C_13_H_28_ were studied by MD simulation. One mixture consists of 100 chains of C_13_H_28_ and 30 chains of C_44_H_90_, and the other mixture consists of 228 chains of C_13_H_28_ and 20 chains of C_150_H_302_, respectively. Both systems were chosen to have almost equal weight fractions in order to focus on the chain length effects on the structural and thermodynamic characteristics of melt films. Table 2 shows the simulation results for the specific volume obtained from NPT MD simulations. The value of the specific volume increases as the temperature increases, and it decreases as the chain becomes longer. The specific volume of the mixture is very close to the average value between those of the corresponding neat components.

The surface tensions of the mixtures estimated at several temperatures are shown in Figure 2 and Table 1. In the case of the less asymmetric mixture in chain length (C_13_H_28_ + C_44_H_90_), the surface tension of the mixture is close to the mean value between the surface tensions of the two neat components. However, for the more asymmetric mixture (C_13_H_28_ + C_150_H_302_), the surface tension exhibits a significant deviation from the mean value between those of the two neat components. It is much closer to the surface tension value of the neat melt of the shorter chain, C_13_H_28_. Such a deviation from a simple mixing behavior indicates that the segment density profile of each component is not uniform along the film thickness direction as discussed below.

**Figure 7:**
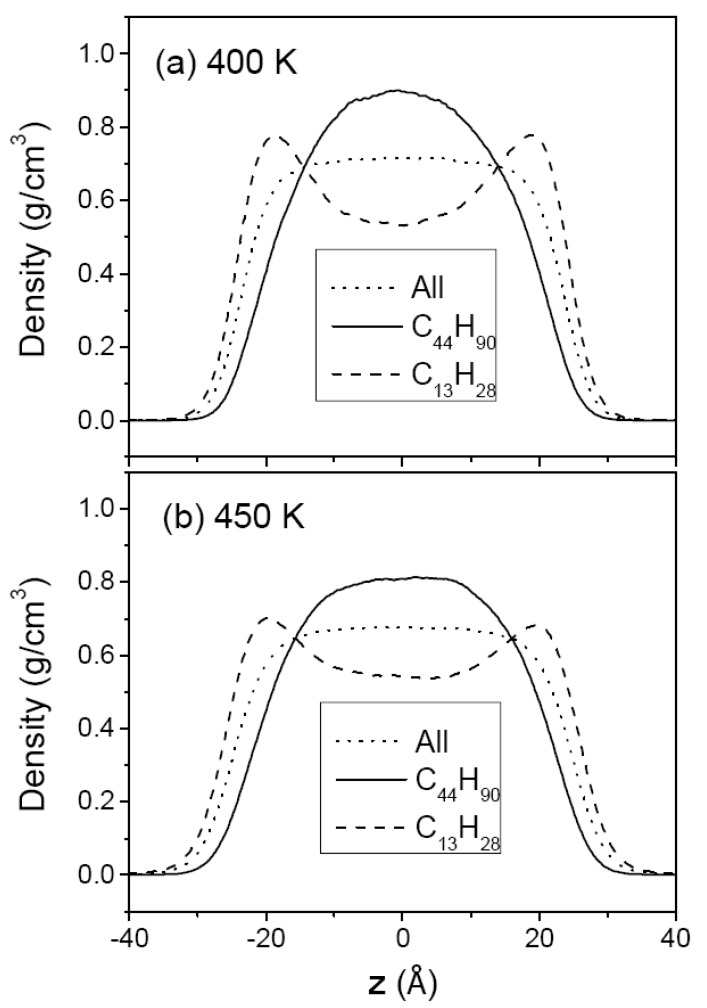
The density profiles of (C_13_H_28_ + C_44_H_90_) mixture melt film at 400 K (**a**) and at 450 K (**b**). The dotted line is the density of all chains, the solid line is the all-atom density profile of C_44_H_90_, and the dashed line is that of C_13_H_28_. The density of each species is normalized to that of all chain atoms; see the caption for Figure 3.

**Figure 8:**
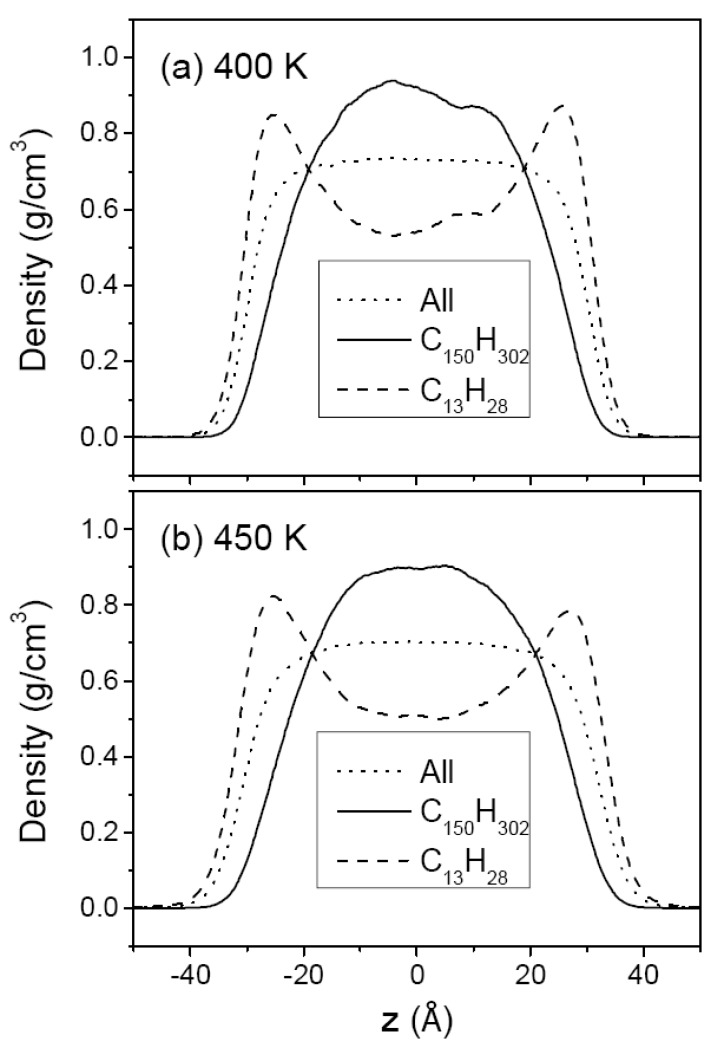
The density profiles of (C_13_H_28_ + C_150_H_302_) mixture melt film at 400 (**a**) and 450 K (**b**). The dotted line is the density of all chains, the solid line is the all-atom density profile of C_150_H_302_, and the dashed line is that of C_13_H_28_. The density of each species is normalized to that of all chain atoms; see the caption for Figure 3.

Figure 7 and Figure 8 show the segment density profiles of the two mixtures, (C_13_H_28_ + C_44_H_90_) and (C_13_H_28_ + C_150_H_302_), respectively. In both Figures, it is noted that the shorter-chain segments are driven to the surface while the longer-chain segments tend to remain in the middle region. This molecular segregation may be explained by either of the enthalpic and the entropic effects. The shorter chains that have lower cohesive energy density in neat liquid would be pushed out to the surface region in order to minimize the total enthalpy. On the other hand, the longer chains with more skeletal C–C bonds would suffer from a larger loss of conformational entropy if they are located at the surfaces [44,45], and hence the longer chains tend to remain in the middle region. From the thermodynamic argument as discussed previouly, the temperature dependence of molecular segregation will reveal which contribution is more dominant for the mixtures of our study.

The temperature dependence of molecular segregation in the mixtures, shown in Figure 7 and Figure 8, is not as obvious as in the case of the chain-end segregation in neat melts. In order to quantify the extent of molecular segregation, we introduce a degree of molecular segregation (DS) which is defined as:

(4) $$\mathrm{DS}=\frac{1}{2}\left(\frac{\int \left|\rho_{1}^{*}(z)-\rho_{o}(z)\right|\;dz+\int \left|\rho_{2}^{*}(z)-\rho_{o}(z)\right|\;dz}{\int \rho_{o}(z)\;dz}\right)$$

where $\rho_{o}$ is the density of all chain atoms and $\rho_{\alpha }^{*}$ is the normalized density of component α ($\rho_{\alpha }^{*}=\frac{\rho_{\alpha }}{w_{\alpha }}$ where $w_{\alpha }$ is the weight fraction of component α). If perfect segregation occurs as in an immiscible blend, DS is 1, and if no segregation occurs, then DS is 0. In a binary mixture of equal weight fraction, Equation (4) reduces to the form:

(5) $$\mathrm{DS}=\frac{\int \left|2\rho_{1}(z)-\rho_{o}(z)\right|\;dz}{\int \rho_{o}(z)\;dz}$$

In the moderately asymmetric mixture (C_13_H_28_ + C_44_H_90_), DS slightly decreases as the temperature increases, with the DS value reduced from 0.22 at 400 K to 0.19 at 450 K, which indicates a greater influence of the enthalpic effects. However, in the highly asymmetric mixture (C_13_H_28_ + C_150_H_302_) the temperature dependence of the DS parameter is reversed: its value of 0.24 is larger than that (0.22) for (C_13_H_28_ + C_44_H_90_) mixture at 400 K, and is further increased to 0.27 at 450 K, becoming much larger than that (0.19) for (C_13_H_28_ + C_44_H_90_) mixture. These trends suggest that in the (C_13_H_28_ + C_44_H_90_) mixture the enthalpic effect which arises from the difference in the cohesive energy density between the two components is more important than the entropic effect which arises from the difference in the number of allowed conformational states under the constraints of the film surface. On the other hand, in the (C_13_H_28_ + C_150_H_302_) mixture the entropic effect becomes greater than the enthalpic effects. In other words, as the chain length of the longer chain increases in a binary mixture of two polymer components of different chain length, the molecular segregation increases due to both the enthalpic and entropic effects, but the entropic effect eventually becomes more important in a mixture containing long enough polymers. In this regard, it seems that the enthalpic effect reaches the upper limit earlier than the entropic effect as a function of chain length.

As mentioned above, the molecular segregation phenomenon becomes more noticeable as the asymmetry in chain length increases. Compared with the case of the (C_13_H_28_ + C_44_H_90_) mixture, C_13_H_28_ molecules in the (C_13_H_28_ + C_150_H_302_) mixture are more favored at the surfaces (See Figure 7 and Figure 8). Hence, the surface tension of the highly asymmetric mixture (C_13_H_28_ + C_150_H_302_) is much closer to the surface tension of the short-chain component than the mean value of both components, as shown in Table 1 and Figure 2. In comparison, the molecular segregation in the moderately asymmetric mixture (C_13_H_28_ + C_44_H_90_) is less pronounced, and accordingly the surface tension of the mixture is closer to the mean value between those of the two neat components.

**Figure 9:**
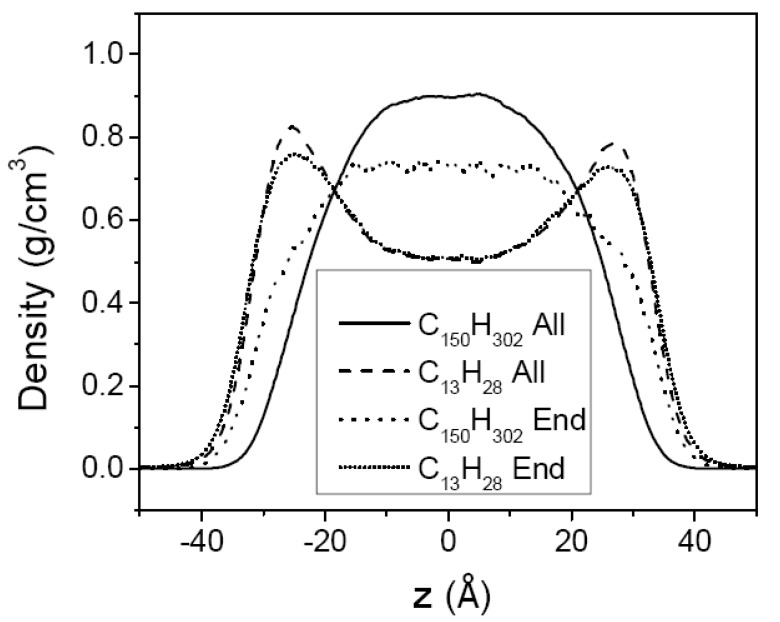
The density profile of methyl end groups of (C_13_H_28_ + C_150_H_302_) mixture melt film at 450 K. The dotted line is the density of methyl groups of C_150_H_302_, and the short-dotted line is that of C_13_H_28_. The all-atom density profile of C_150_H_302_ (solid line) and that of C_13_H_28_ (dashed line) are shown for comparison. The density of each species is normalized to that of all chain atoms; see the caption for Figure 3.

The density profiles of the methyl chain-end groups in the mixtures are shown in Figure 9 for the highly asymmetric mixture (C_13_H_28_ + C_150_H_302_) at 450 K. In pure melts, the effect of the end group segregation at the surface becomes more pronounced for longer chains. In mixtures, however, the methyl groups of shorter chains are more segregated to the surface region than those of longer chains based on the normalized methyl group densities. This reflects the fact that the longer chains overall are pushed away from the surface region. However, relative to the corresponding all-atom density of the longer chain alone, the normalized density profile of methyl chain ends of the longer chain is more strongly enhanced in the surface region, again demonstrating a strong driving force to favor the chain ends in the surface.

**Figure 10:**
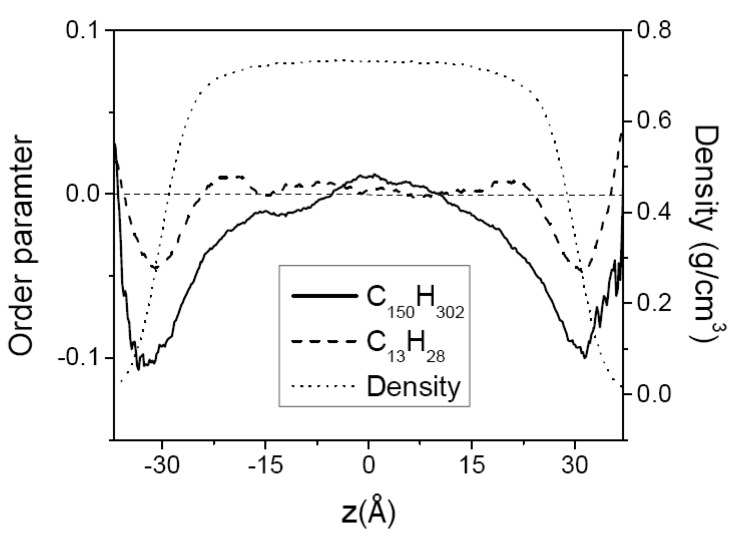
The order parameters of each component of (C_150_H_302_ + C_13_H_28_) mixture melt film at 400 K along the film thickness direction (*z*) with the film center at *z* = 0, plotted together with the overall density profile.

In Figure 10 the orientational order parameter of (C_150_H_302_ + C_13_H_28_) mixture in the thin film at 400 K is shown. Similar to the case of the neat melts, the chain-segment vectors in the mixture tend to be aligned parallel to the surface in the film surfaces. The longer chains exhibit a higher orientational order than the shorter chains. However, the chain-segment order of C_13_H_28_ in the mixture is much stronger at the surface than in the neat melts, with its value almost twice the value of the pure melt, most likely due to the molecular segregation/confinement of the short chains to the narrow surface region. Doruker previously reported similar results from Monte Carlo simulation [46]. At 400 K the value of the orientational order of C_150_H_302_ in the mixture is slightly lower than that in the pure melt shown in Figure 6, but the orientational order of longer chains along the film surface extends into the film deeper than in neat melts, probably caused by the molecular confinement in the region away from the surface.

## 4. Conclusions

Molecular dynamics simulations on thin melt films comprised of neat polyethylenes (C_44_H_90_ and C_150_H_302_) and their mixtures with C_13_H_28_ (C_13_H_28_ + C_44_H_90_ and C_13_H_28_ + C_150_H_302_) were performed by using a united atom model. We obtained surface tension values from the atomic virial tensor, and the estimated values are in good agreement with experimental data within 10%–15% deviation. Compared to the case of C_13_H_28_ melt films, we observed a greater extent of surface segregation of methyl chain ends for the longer PE chains studied here: C_44_H_90_ and C_150_H_302_. At lower temperatures the methyl groups are more strongly segregated to the surface, indicating that the chain-end segregation in neat PE melt films is attributed mainly to the enthalpy effect of pushing out the lower cohesive-energy-density methyl group to the free surface. In binary mixture melts, the short chains are more likely to be found in the surface than the long chains. A careful analysis of the dependence of this molecular segregation on the temperature indicates that the segregation of the shorter chains in the film surface is due to not only the enthalpic effects caused by the difference in the cohesive energy density between the two components but also the entropic effects arising from the difference in the allowed chain conformational states in the film surface. It was found that the molecular segregation in the moderately asymmetric mixture (C_13_H_28_ + C_44_H_90_) is more dominated by the enthalpic effect whereas the segregation is more strongly influenced by the entropic effects in the highly asymmetric mixture (C_13_H_28_ + C_150_H_3020_). The estimated surface tension values of the mixtures are consistent with the observed molecular segregation characteristics. Despite this molecular segregation, the normalized density of methyl chain ends of the longer chain is more strongly enhanced, as compared with the all-atom density of the longer chain itself, in the surface region of melt mixtures. In addition, the molecular segregation results in higher order parameters of shorter-chain segments at the surface and deeper persistence of surface-induced segmental order into the film for the longer chains, as compared with those in neat melt films.

## Figures and Tables

**Table 1:** The surface tension of polyethylene films obtained from molecular dynamics simulations. The unit is dyn/cm.

Temp. (K)	C_13_H_28_		C_44_H_90_		C_150_H_302_		Mixture C_13_ + C_44_ C_13_ + C_150_	
	Sim. ^a^	Exp. ^b^	Sim.	Exp. ^c^	Sim.	Exp. ^d^	Sim.	Sim.
300	29.9	25.4	…	…	…	…	…	…
350	24.2	21.0	30.6	27.8	…	…	27.9	…
400	19.1	…	27.9	…	30.7	27.4	23.4	23.9
450	…	…	24.5	…	28.6	24.1	18.5	19.4
500	…	…	…	…	24.8	…	…	…

^a^ Taken from Chang et al. [3], ^b^ reference [36], ^c^ from extrapolation using data in reference [36], ^d^ reference [37].

**Table 2:** The specific volume of neat polyethylene melts and their mixtures at various temperatures, obtained from molecular dynamics simulations. The unit is cm^3^/gr.

Temp. (K)	Pure Melt			Mixture (50:50)	
	C_13_H_28_^a^	C_44_H_90_	C_150_H_302_	C_13_ + C_44_	C_13_ + C_150_
350	1.383	1.232		1.300	
400	1.443	1.269	1.233	1.357	1.335
450	1.520		1.274		1.398

^a^ Taken from Chang et al. [3].

## References

[1] Mansfield K.F., Theodorou D.N. (1991). Molecular dynamics simulation of a glassy polymer surface. Macromolecules.

[2] Harris J.G. (1992). Liquid-vapor interfaces of alkane oligomers: Structure and thermodynamics from molecular dynamics simulations of chemically realistic models. J. Phys. Chem..

[3] Chang J., Han J., Yang L., Jaffe R.L., Yoon D.Y. (2001). Structure and properties of polymethylene melt surfaces from molecular dynamics simulations. J. Chem. Phys..

[4] Harmandaris V.A., Daoulas K.C., Mavrantzas V.G. (2005). Molecular dynamics simulation of a polymer melts/solid interface: Local dynamics and chain mobility in a thin film of polyethylene melt adsorbed on graphite. Macromolecules.

[5] Peter S., Meyer H., Baschnagel J. (2009). Molecular dynamics simulations of concentrated polymer solutions in thin film geometry. I. Equilibrium properties near the glass transition. J. Chem. Phys..

[6] Peter S., Meyer H., Baschnagel J. (2009). Molecular dynamics simulations of concentrated polymer solutions in thin film geometry. II. Solvent evaporation near the glass transition. J. Chem. Phys..

[7] Torres J.A., Nealey P.F., de Pablo J.J. (2000). Molecular simulation of ultrathin polymeric films near the glass transition. Phys. Rev. Lett..

[8] Morita H., Tanaka K., Kajiyama T., Nishi T., Doi M. (2006). Study of the glass transition temperature of polymer surface by coarse-grained molecular dynamics simulation. Macromolecules.

[9] Xia W., Hsu D.D., Keten S. (2015). Molecular weight effects on the glass transition and confinement behavior of polymer thin films. Macromol. Rapid Commun..

[10] Varnik F., Baschnagel J., Binder K. (2002). Reduction of the glass transition temperature in polymer films: A molecular-dynamics study. Phys. Rev. E.

[11] Varnik F., Baschnagel J., Binder K. (2000). Molecular dynamics results on the pressure tensor of polymer films. J. Chem. Phys..

[12] Kumar N., Manik G. (2016). Molecular dynamics simulations of polyvinyl acetate-perfluorooctane based anti-stain coating. Polymer.

[13] Eslami H., Müller-Plathe F. (2013). How thick is the interphase in an ultrathin polymer film? Coase-grained molecular dynamics simulations of polyamide-6,6 on graphene. J. Phys. Chem. C.

[14] Glynos E., Johnson K.J., Frieberg B., Chremos A., Narayanan S., Sakellariou G., Green P.F. (2017). Free surface relaxations of star-shaped polymer films. Phys. Rev. Lett..

[15] Solar M., Binder K., Paul W. (2017). Relaxation processes and glass transition of confined polymer melts: A molecular dynamics simulation of 1,4-polybutadiene between graphite walls. J. Chem. Phys..

[16] Jacobs M., Liang H., Pugnet B., Dobrynin A.V. (2018). Molecular dynamics simulations of surface and interfacial of graft polymer melts. Langmuir.

[17] Lyulin A.V., Balabaev N.K., Baljon A.R.C., Mendoza G., Frank C.W., Yoon D.Y. (2017). Interfacial and topological effects on the glass transition in free-standing polystyrene films. J. Chem. Phys..

[18] Lee S., Lyulin A.V., Frank C.W., Yoon D.Y. (2017). Interface characteristics of polystyrene melts in free-standing thin films and on graphite surface from molecular dynamics simulations. Polymer.

[19] Li S., Ding M., Shi T. (2017). Effect of bidispersity on structure and entanglement of confined polymer films. J. Phys. Chem. B.

[20] Li S., Chen Q., Ding M., Shi T. (2017). Effect of bidispersity on dynamics of confined polymer films. Polymers.

[21] Xia W., Lan T. (2019). Interfacial dynamics governs the mechanical properties of glass polymer thin films. Macromolecules.

[22] Sorensen R.A., Liau W.B., Kesner L., Boyd R.H. (1988). Prediction of polymer crystal structures and properties: Polyethylene and poly(oxymethylene). Macromolecules.

[23] Smith G.D., Yoon D.Y. (1994). Equilibrium and dynamics properties of polymethylene melts from molecular dynamics simulations. I. *n*-Tridecane. J. Chem. Phys..

[24] Jorgensen W.L., Maxwell D.S., Tirado-Rives J. (1996). Development and testing of the OPLS all-atom force field on conformational energetics and properties of organic liquids. J. Am. Chem. Soc..

[25] Chen B., Siepmann J.I. (1999). Tranferable potential for phase equilibria. 3. Explicit-hydrogen description of normal alkane. J. Phys. Chem. B.

[26] Chang J., Sandler S.I. (2004). Interatomic Lennard-Jones potentials of linear and branched alkanes calibrated by Gibbs ensemble simulations for vapor-liquid equilibria. J. Chem. Phys..

[27] Jorgensen W.L., Madura J.D., Swenson C.J. (1984). Optimized intermolecular potential functions for liquid hydrocarbons. J. Am. Chem. Soc..

[28] Nath S.K., Escobedo F.A., de Pablo J.J. (1998). On the simulation of vapor-liquid equilibria for alkanes. J. Chem. Phys..

[29] Martin M.G., Siepmann J.I. (1998). Transferable potentials for phase equilibria. 1. United-atom description of n-alkanes. J. Phys. Chem. B.

[30] Paul W., Yoon D.Y., Smith G.D. (1995). An optimized united atom model for simulations of polymethylene melts. J. Chem. Phys..

[31] Nosé S. (1984). A unified formulation of the constant temperature molecular dynamics methods. J. Chem. Phys..

[32] Allen M.P., Tildesley D.J. (1990). Computer Simulation of Liquids.

[33] Kirkwood J.G., Buff F.P. (1949). The statistical mechanical theory of surface tension. J. Chem. Phys..

[34] Rowlinson J.S., Widom B. (1982). Molecular Theory of Capillarity.

[35] Mecke M., Winkelmann J., Fisher J. (1997). Molecular dynamics simulation of the liquid-vapor interface: The Lennard-Jones fluid. J. Chem. Phys..

[36] Thermodynamics Research Center (1990). TRC Thermodynamic Tables, Hydrocarbons.

[37] Dee G., Sauer B.B. (1992). The molecular weight and temperature dependence of polymer surface tension: Comparison of experiment with interface gradient theory. J. Colloid Interface Sci..

[38] Doruker P., Mattice W.L. (1999). Mobility of the surface and interior of thin films composed of amorphous polyethylene. Macromolecules.

[39] Yethiraj A. (1995). Entropic and enthalpic surface segregation from blends of branched and linear polymers. Phys. Rev. Lett..

[40] Scheutjens J.M.H.M., Fleer G.J. (1979). Statistical theory of the adsorption of interacting chain molecules. 1. Partition function, segment density distribution, and adsorption isotherms. J. Phys. Chem..

[41] Theodorou D.N. (1988). Microscopic structure and thermodynamic properties of bulk copolymers and surface-active polymers at interfaces. 1. Theory. Macromolecules.

[42] Klein J., Kerle T., Zink F., Eiser E. (2000). Segmental interaction parameters of binary polymer mixtures evaluated from binodals and from surface-segregation profiles: Comparison with small-angle neutron scattering. Macromolecules.

[43] Barton A.F.M. (1983). Handbook of Solubility Parameters and Other Cohesion Parameters.

[44] Hariharan A., Kumar S.K., Russell T.P. (1990). A lattice model for the surface segregation of polymer chains due to molecular weight effects. Macromolecules.

[45] Van der Gucht J., Besseling N.A.M., Fleer G.J. (2002). Surface segregation in polydisperse polymer melts. Macromolecules.

[46] Doruker P. (2002). Simulation of polyethylene thin films composed of various chain lengths. Polymer.

